# EHMTI-0393. Abnormal ictal large-scale network connectivity in migraine without aura: a resting-state functional connectivity study

**DOI:** 10.1186/1129-2377-15-S1-K1

**Published:** 2014-09-18

**Authors:** FM Amin, A Hougaard, S Magon, MS Asghar, NN Ahmad, E Rostrup, T Sprenger, M Ashina

**Affiliations:** 1 Danish Headache Center, Glostrup Hospital, Glostrup, Denmark; 2 Department of Neurology, University Hospital Basel, Basel, Switzerland; 3 Functional Imaging Unit, Glostrup Hospital, Glostrup, Denmark

## Introduction

Alterations in cerebral resting-state functional connectivity (RSFC) have been reported outside of migraine attacks. To date, no studies studied possible changes in RSFC during migraine attacks.

## Aims

To investigate resting-state functional connectivity in salience (SN), sensorimotor (SMN) and default mode networks (DMN) during the early phase of pituitary adenylate cyclase-activating polypeptide-38 (PACAP38)-induced migraine attacks.

## Methods

In a double-blind randomized study, 24 female migraine patients without aura received intravenous PACAP38 or vasoactive intestinal polypeptide (VIP) for 20 min. Both peptides are closely related and cause vasodilatation, but only PACAP38 induces migraine attacks. VIP was therefore used as an active placebo. Functional MRI was recorded before and during PACAP38-induced attacks (n=16) and before and after VIP infusion (n=15). Data were analyzed by SPM8 and the REST toolbox for Matlab in a seed-based fashion.

## Results

During PACAP38-induced attacks, we found increased connectivity of the bilateral opercular part of the inferior frontal gyrus (Brodmann area 44) in the SN. In SMN, there was increased connectivity in the right premotor cortex and decreased activity in the left visual cortex. Several areas showed increased (left primary auditory, secondary somatosensory, premotor and visual cortices, and left superior longitudinal fascicle) and decreased (right cerebellum and left frontal lobe) connectivity in DMN. We found no resting-state network changes after VIP.

## Conclusion

The early phases of PACAP38-induced migraine attacks are associated with altered connectivity of several large-scale functional networks of the brain.

